# Pectoralis Major Myocutaneous Flap for Chest Wall Reconstruction After Dermatofibrosarcoma Protuberans Resection: A Case Report

**DOI:** 10.7759/cureus.107385

**Published:** 2026-04-20

**Authors:** Tania Y Villa-Olivares, Carina M Álvarez-Dávalos, Hector Alvarez-Trejo, Quitzia L Torres-Salazar

**Affiliations:** 1 General Medicine, Centro Universitario del Sur, Universidad de Guadalajara, Guadalajara, MEX; 2 Surgery, Centro Universitario de Ciencias de la Salud de la Universidad de Guadalajara, Guadalajara, MEX; 3 Plastic Surgery, Universidad de Guadalajara, Guadalajara, MEX; 4 Biomedical Sciences, Universidad Juárez del Estado de Durango, Durango, MEX

**Keywords:** chest wall reconstruction, dermatofibrosarcoma protuberans, pectoralis major flap, soft tissue sarcoma, wide local excision

## Abstract

Dermatofibrosarcoma protuberans (DFSP) is a rare cutaneous soft tissue sarcoma characterized by slow growth but significant local aggressiveness and a high risk of recurrence when inadequately excised. Its clinical presentation may mimic benign lesions, frequently leading to delayed diagnosis and suboptimal initial management. Achieving complete surgical resection with negative margins remains the cornerstone of treatment; however, wide excision often results in complex soft tissue defects that require appropriate reconstructive strategies.

We present the case of a 49-year-old man with a recurrent infraclavicular lesion initially misdiagnosed as a keloid and treated without histopathological confirmation. Following recurrence, a biopsy established the diagnosis of DFSP. The patient underwent wide local excision (WLE) with oncologic margins, resulting in a full-thickness defect measuring approximately 6 cm in diameter. Immediate reconstruction was performed using a pedicled pectoralis major myocutaneous flap designed to provide reliable vascularized coverage and allow tension-free closure.

Histopathological examination confirmed DFSP with negative surgical margins; the closest margin measured 0.5 cm. The postoperative course was uneventful, and the patient remains free of local recurrence or metastasis after four years of follow-up.

This case highlights the importance of the early histopathological evaluation of atypical cutaneous lesions and underscores the role of reconstructive surgery as an integral component of oncologic management. In selected cases, pedicled myocutaneous flaps provide a reliable and effective option for immediate reconstruction, enabling adequate tumor resection while preserving functional and aesthetic outcomes.

## Introduction

Dermatofibrosarcoma protuberans (DFSP) is an uncommon cutaneous soft tissue sarcoma, accounting for approximately 0.1% of all malignant neoplasms and between 1.8% and 6% of soft tissue sarcomas worldwide. Its estimated annual incidence ranges from 0.8 to five cases per million inhabitants and predominantly affects adults in the third to fifth decades of life [1]. Despite its low metastatic potential (reported in less than 5% of cases), DFSP is characterized by a marked propensity for local aggressiveness and recurrence, with rates historically ranging from 10% to 50% and even reaching 26%-60% in some series following inadequate excision [2]. This high recurrence rate is largely attributable to frequent initial mismanagement, as DFSP is often clinically misdiagnosed as a benign lesion and treated without histopathological confirmation or with insufficient surgical margins. In this context, the present case illustrates this diagnostic and therapeutic challenge, emphasizing the importance of early histopathological evaluation and adequate oncologic resection to achieve durable local control.

From a biological standpoint, DFSP originates in the dermis and demonstrates a distinctive infiltrative growth pattern, with extension into the subcutaneous tissue, fascia, and occasionally deeper structures. This behavior is largely driven by the characteristic chromosomal translocation t(17;22)(q22;q13), leading to *COL1A1*-*PDGFB* fusion and the constitutive activation of the platelet-derived growth factor pathway, which promotes tumor proliferation and local invasion [3]. Clinically, its indolent evolution often results in delayed diagnosis, as lesions may initially appear as innocuous cutaneous thickening, allowing progressive infiltration over months or even years before appropriate treatment is established. This biological behavior not only explains the high rates of recurrence but also translates into complex surgical challenges, particularly when wide excision results in significant soft tissue defects requiring reconstruction. This combination of low metastatic potential but high local recurrence represents the central paradox of DFSP and defines its surgical challenge [4].

Achieving oncologic control depends fundamentally on complete excision with histologically negative margins [5]. However, due to its tentacle-like subclinical extensions, inadequate resection margins remain the principal cause of recurrence [6]. Conventional recommendations suggest margins of 2-3 cm, which, although effective in reducing recurrence, may result in significant soft tissue defects, particularly in anatomically complex or aesthetically sensitive regions. These defects frequently require reconstructive strategies that ensure reliable coverage while preserving contour and function [7].

Consequently, the management of DFSP lies at the intersection of oncologic radicality and reconstructive precision. Mohs micrographic surgery (MMS) has been increasingly adopted due to its ability to achieve comprehensive margin assessment and has been associated with lower reported recurrence rates in some series [8]. However, its superiority over wide local excision (WLE) remains a subject of debate, particularly due to the lack of high-quality comparative studies [9]. Importantly, both MMS and WLE share a common consequence: the creation of large, complex surgical defects, particularly in anatomically challenging regions. Therefore, immediate reconstruction should not be regarded merely as a restorative step but rather as an integral component of the surgical strategy, enabling adequate oncologic resection while optimizing functional and aesthetic outcomes.

Given these challenges, the role of the reconstructive surgeon becomes critical, serving not only to restore form and function but also to facilitate oncologically sound resection without compromise. The present report aims to describe a surgical strategy that integrates oncologic principles with a structured reconstructive approach using a pedicled pectoralis major myocutaneous flap, highlighting its applicability, reproducibility, and long-term oncologic safety in the management of DFSP. This case was reported in accordance with the Surgical CAse REport (SCARE) guidelines [10].

## Case presentation

A 49-year-old male patient with no significant past medical history presented with a progressively enlarging cutaneous lesion located in the right infraclavicular region. The lesion had initially appeared approximately nine months prior to consultation as a small nodular thickening of the skin. It was initially evaluated by another physician and clinically diagnosed as a keloid scar, leading to surgical excision without prior histopathological assessment. The patient experienced early local recurrence approximately three months after the initial procedure, with progressive enlargement of the lesion thereafter.

Six months after recurrence, the patient sought further evaluation at our institution due to persistent growth of the lesion accompanied by significant pruritus and local discomfort. Physical examination revealed a firm, nodular cutaneous mass in the right infraclavicular region, measuring approximately 4.5 cm in diameter, clinically well-defined but adherent to deeper planes. The lesion exhibited a light brown coloration, was slightly raised, and had intact overlying skin without ulceration. It demonstrated limited mobility, consistent with involvement of the underlying subcutaneous tissue. The patient reported progressive enlargement, with an approximate increase of 1 cm in diameter over a six-month period following the initial excision. No regional lymphadenopathy was detected. A detailed chronological summary of the clinical course, diagnostic workup, and surgical management is provided in the timeline table (Table 1).

**Table 1:** Chronological timeline of clinical evolution, diagnostic assessment, and surgical management. The table outlines the progression of the lesion from initial presentation to definitive treatment, including misdiagnosis, recurrence, histopathological confirmation, reconstructive intervention, and long-term oncologic outcome. DFSP, dermatofibrosarcoma protuberans; FNCLCC, French Federation of Cancer Centers Sarcoma Group

Time point	Clinical event
9 months before presentation	The patient noticed a small nodular thickening in the right infraclavicular region
Initial medical evaluation	Lesion clinically diagnosed as a keloid scar at another institution
Initial treatment	Surgical excision performed without prior histopathological analysis
3 months post initial surgery	Early local recurrence of the lesion, with progressive increase in size
6 months after recurrence	The patient presented to our institution with persistent growth, pruritus, and discomfort
Second clinical evaluation	Physical examination revealed a 4.5 cm firm, nodular, well-circumscribed lesion; no lymphadenopathy detected
Diagnostic procedure	Incisional biopsy performed
Histopathological diagnosis	Dermatofibrosarcoma protuberans confirmed; no immunohistochemistry due to financial constraints
Multidisciplinary decision	Indication for wide local excision with immediate reconstruction
Surgical intervention	Wide local excision with 1 cm circumferential and 1.5 cm deep margins performed, resulting in a 6 cm full-thickness defect
Reconstruction planning	Design of pedicled pectoralis major myocutaneous flap with inferolateral skin paddle
Reconstructive procedure	Subfascial elevation of flap preserving thoracoacromial pedicle; rotation-advancement transposition and tension-free closure achieved
Histopathological findings (final)	DFSP, FNCLCC grade 1; tumor size: 6 × 4.5 × 3.5 cm; margins free (closest margin: 0.5 cm)
Postoperative course	Uneventful; no complications (necrosis, infection, and dehiscence)
Follow-up	4-year follow-up with no evidence of local recurrence or metastasis

Given the atypical clinical course and rapid recurrence after prior excision, the initial differential diagnosis included benign fibroproliferative lesions such as keloid, hypertrophic scar, and dermatofibroma. An incisional biopsy was performed. Histopathological analysis revealed a spindle cell neoplasm with features consistent with DFSP. Immunohistochemical studies were not performed due to financial constraints.

**Figure 1:**
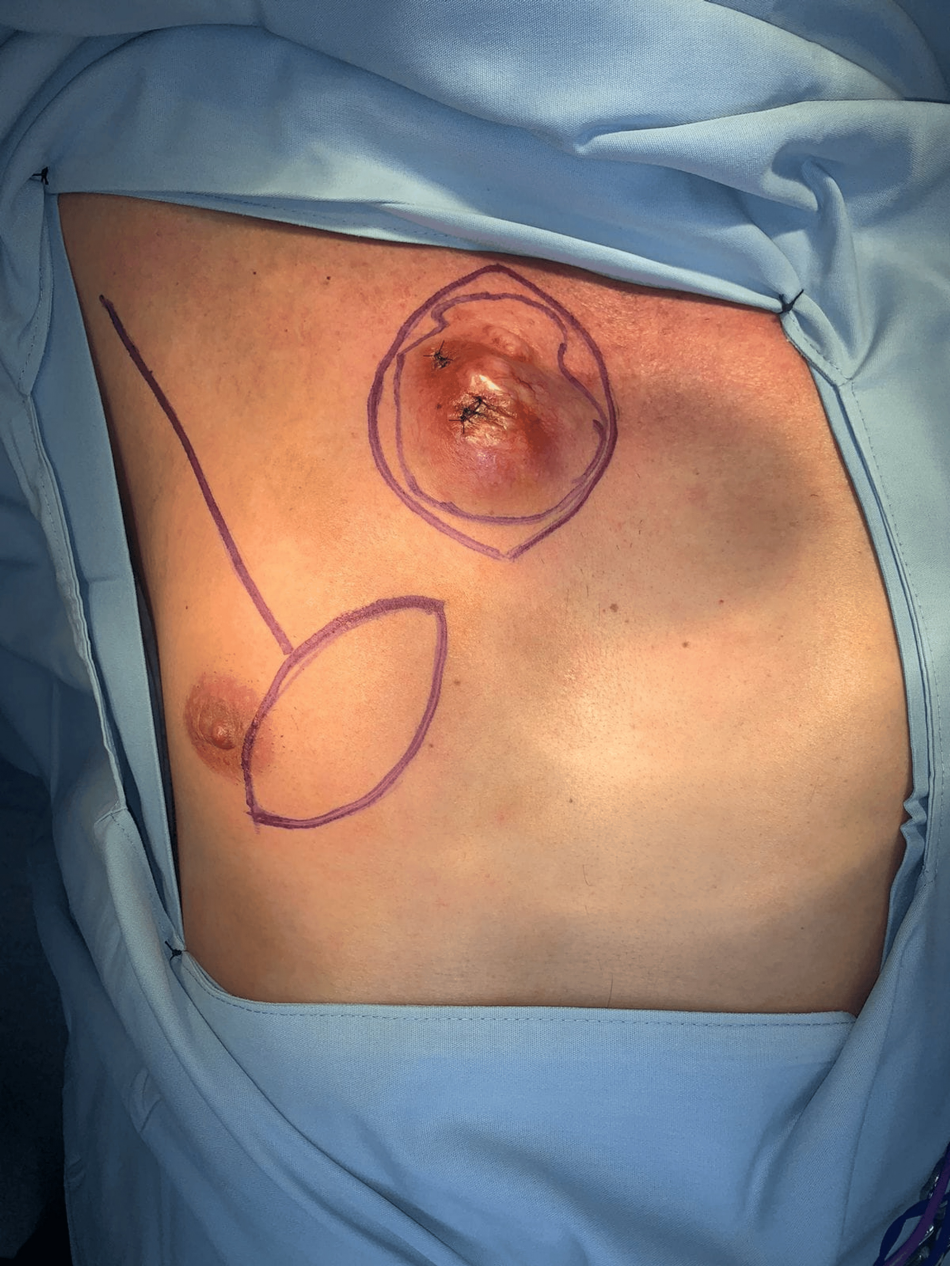
Preoperative clinical appearance and surgical planning. The central nodular lesion in the right infraclavicular region is outlined by the innermost marking. The surrounding outer marking indicates the planned oncologic excision margin. Inferolateral to the lesion, an elliptical skin paddle is designed over the pectoralis major muscle, corresponding to the planned pedicled myocutaneous flap. Its orientation reflects the intended arc of transposition toward the infraclavicular defect and the anticipated course of the flap from the lateral chest wall to the recipient site.

After multidisciplinary evaluation, the patient was scheduled for definitive surgical management. A wide local excision was performed with oncologic margins of 1 cm circumferentially and 1.5 cm in depth. This resulted in a circular full-thickness cutaneous defect measuring approximately 6 cm in diameter in the right infraclavicular region (Figure 1).

The surgical specimen consisted of an en bloc resection of the skin and underlying soft tissues measuring 9.5 × 6.5 × 5.5 cm. The skin surface was light brown and irregular, with nodular areas and two prior surgical scars measuring 0.6 cm and 1 cm. The deep aspect of the specimen included skeletal muscle fibers.

On sectioning, a solid tumor measuring 6 × 4.5 × 3.5 cm was identified. The lesion was light brown and semi-firm in consistency and showed relatively well-defined borders. It infiltrated the subcutaneous adipose tissue without the involvement of the underlying skeletal muscle.

Given the size and location of the defect, a pedicled pectoralis major myocutaneous flap was planned preoperatively. The skin paddle was designed in an inferolateral position relative to the defect, over the pectoralis major muscle, following the orientation of its muscle fibers to optimize vascular reliability (Figure 1). The dimensions of the skin island were tailored to match the approximately 6 cm circular defect.

**Figure 2:**
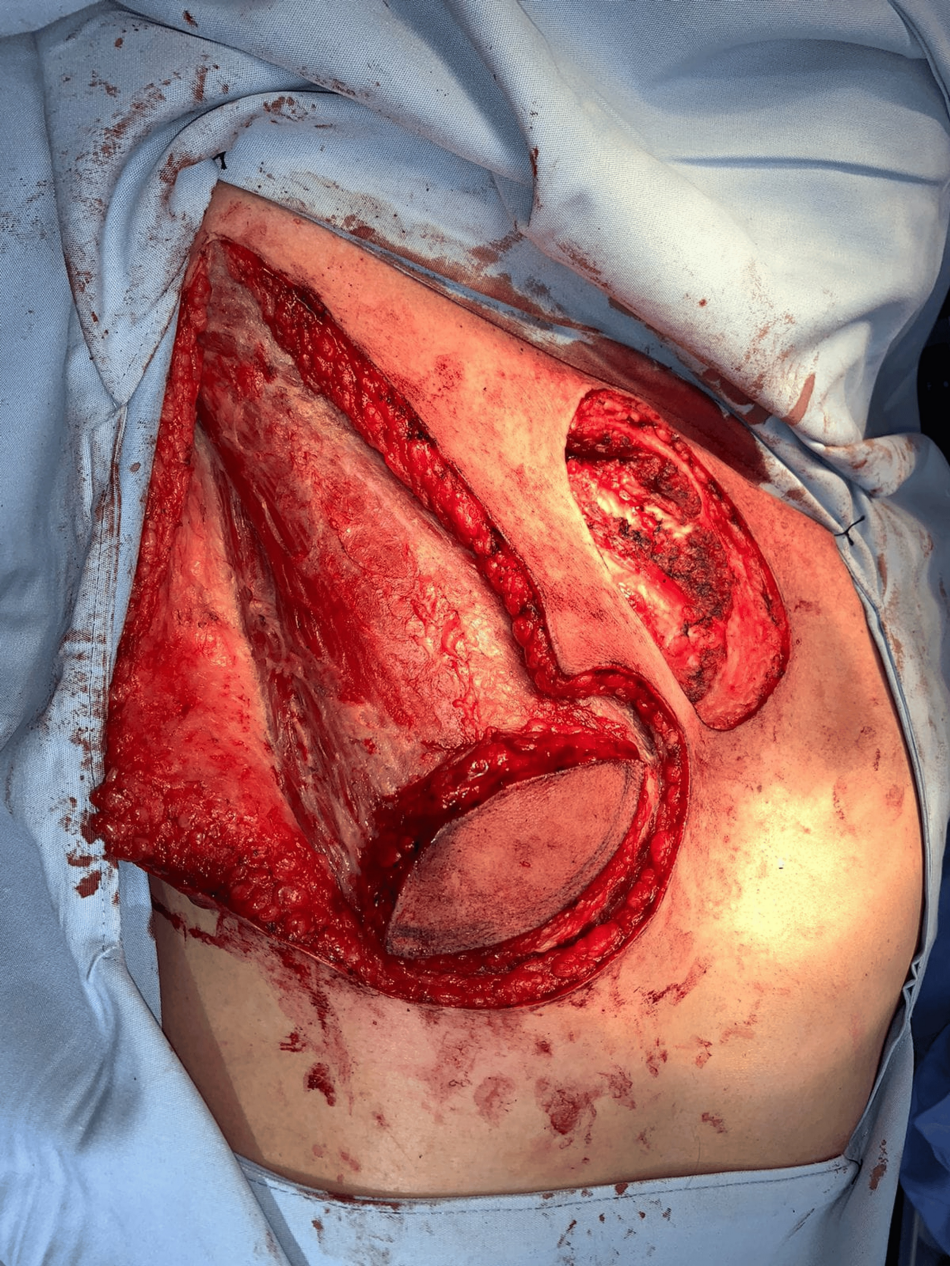
Intraoperative defect following wide local excision. A full-thickness circular defect measuring approximately 6 cm in diameter is observed in the right infraclavicular region after tumor resection, with the exposure of the underlying pectoralis major muscle and surrounding subcutaneous tissue. The adjacent elevated flap can be partially appreciated, demonstrating the plane of dissection and the relationship between the defect and the planned reconstructive tissue. This view highlights the extent of the defect and the need for well-vascularized tissue to achieve adequate coverage.

Following tumor resection, a full-thickness infraclavicular defect was created, with the exposure of subcutaneous tissue and the preservation of the underlying muscle (Figure 2).

**Figure 3:**
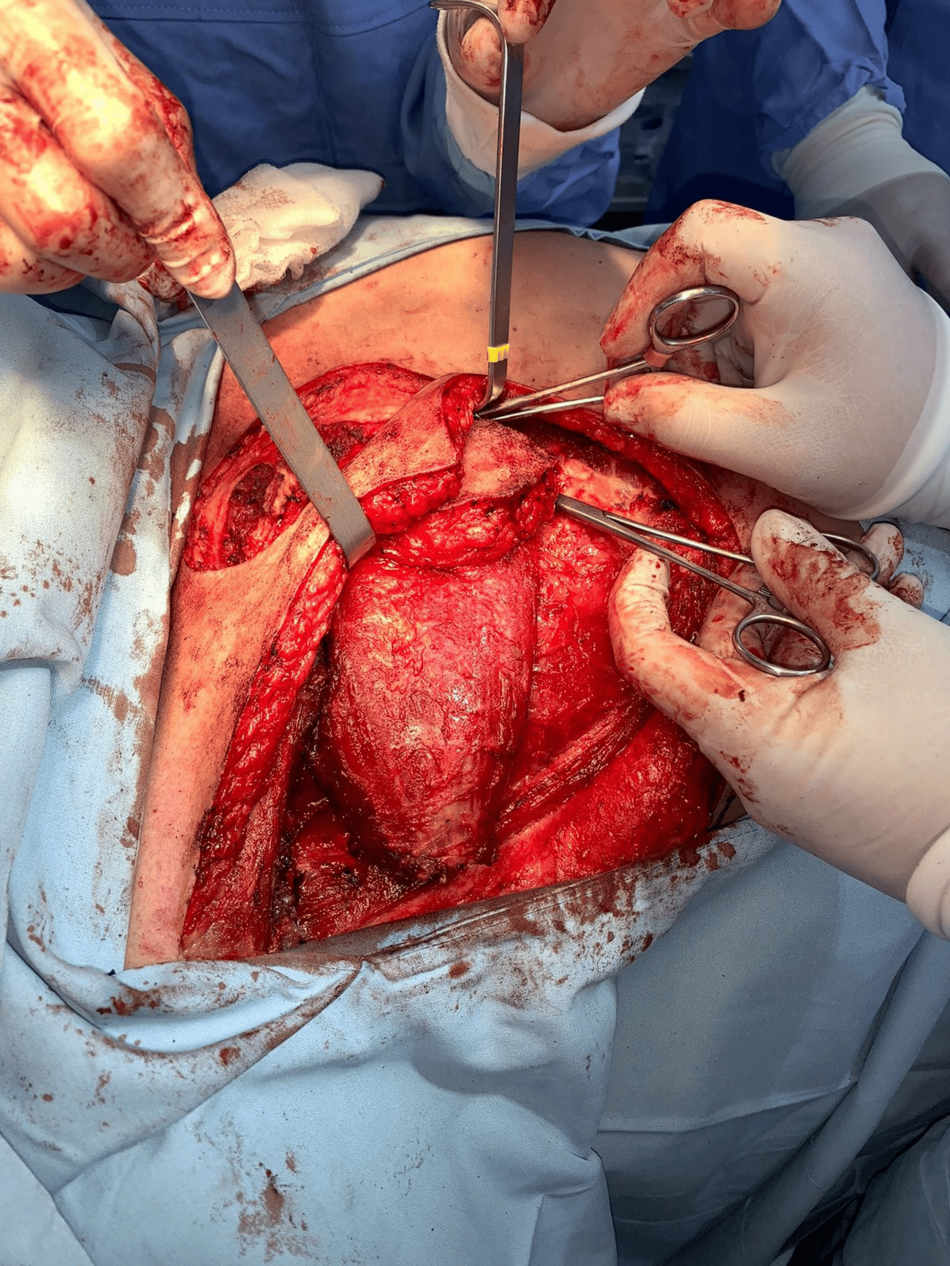
Flap elevation and mobilization. The pedicled pectoralis major myocutaneous flap is elevated in a subfascial plane, incorporating a segment of the muscle while preserving the thoracoacromial vascular pedicle. The pectoralis major muscle is clearly exposed, demonstrating the plane of dissection, while the overlying skin paddle remains attached to the underlying muscle. The flap is shown partially mobilized, illustrating its arc of rotation toward the recipient defect and the preservation of its vascular supply.

The flap was elevated in a subfascial plane, incorporating a segment of the pectoralis major muscle. Careful dissection was performed to preserve the thoracoacromial vascular pedicle as the dominant blood supply. The muscle was mobilized toward its vascular base, allowing an adequate arc of rotation (Figure 3).

**Figure 4:**
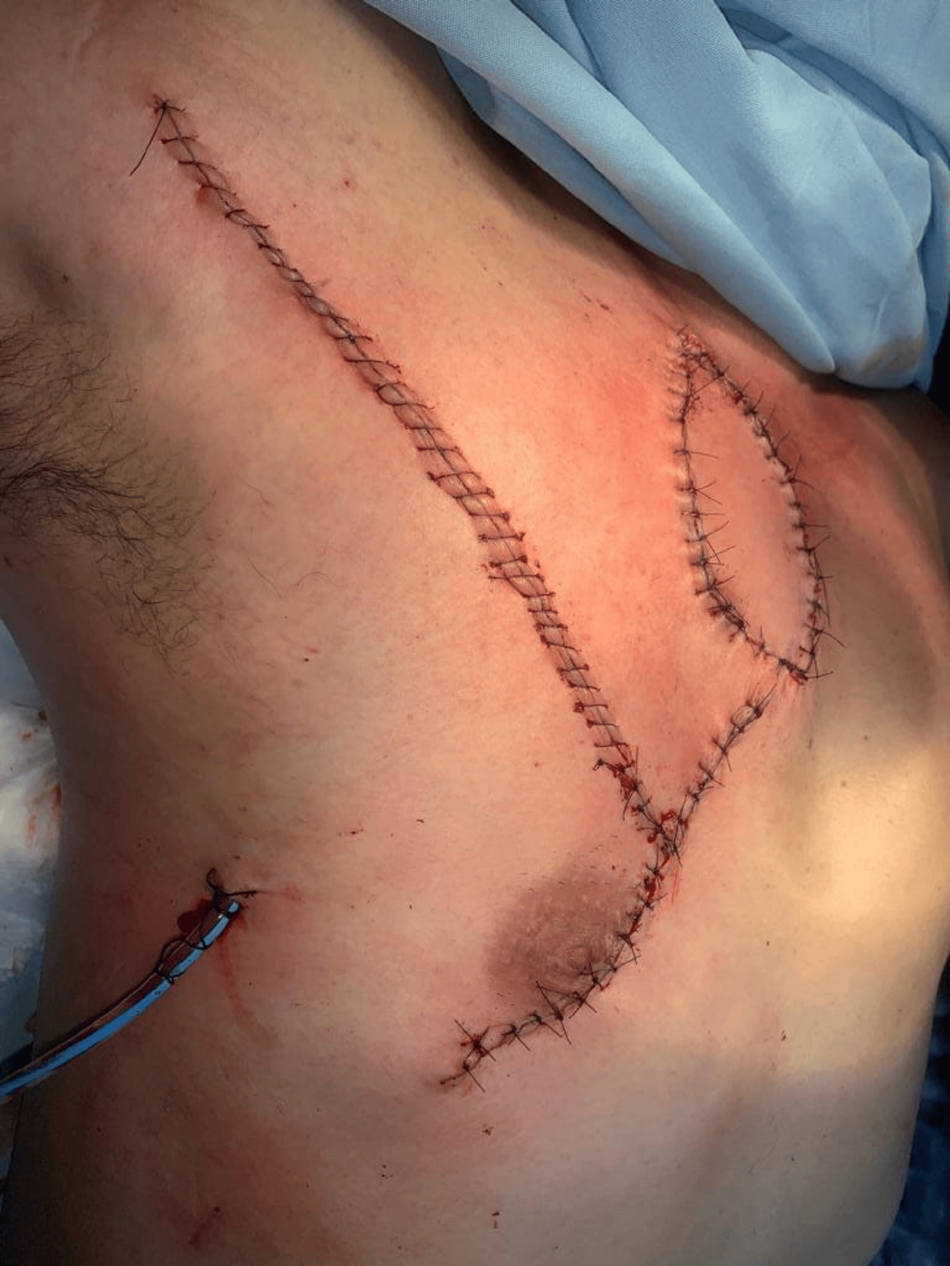
Immediate postoperative result. The flap has been transposed using a combined rotation and advancement technique, achieving the complete coverage of the defect. The skin paddle demonstrates adequate perfusion, with preserved color and no signs of vascular compromise. The primary closure of the donor site is observed, with a closed-suction drain in place and appropriate contour restoration, reflecting balanced tension distribution across the surgical site.

The flap was then transposed to the defect using a combined rotation and advancement maneuver, ensuring tension-free coverage while avoiding pedicle torsion or compression. The skin island was inset without redundancy, and the adequate perfusion of both the muscular and cutaneous components was confirmed intraoperatively. The donor site was closed primarily after tissue mobilization, without the need for grafting or additional releasing incisions. Final closure demonstrated the appropriate contour and coverage of the defect, with the satisfactory distribution of tension across both recipient and donor sites (Figure 4).

Histopathological examination demonstrated a malignant mesenchymal neoplasm located beneath a stratified squamous epithelium with preserved maturation. The tumor was composed of spindle-shaped fibroblastic cells arranged in a characteristic storiform pattern, with poorly defined and infiltrative margins. It infiltrated the subcutaneous tissue without extension into the underlying skeletal muscle. The surrounding stroma showed edematous changes, and adnexal structures were preserved.

**Figure 5:**
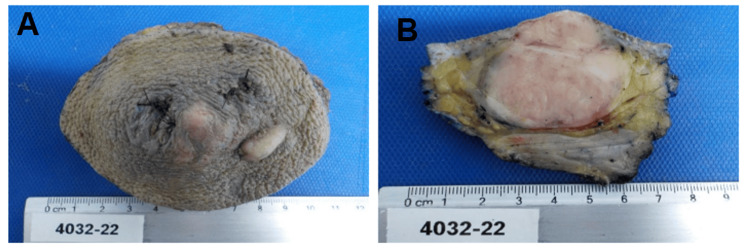
Macroscopic appearance of the surgical specimen. (A) External view showing an en bloc skin and soft tissue resection with irregular surface and nodular areas corresponding to the lesion. The specimen includes the overlying skin and subcutaneous tissue, consistent with wide local excision margins. (B) Sectioned specimen demonstrating a well-defined, solid tumor with a pale, homogeneous cut surface infiltrating the subcutaneous tissue without the involvement of the underlying skeletal muscle. These macroscopic features are consistent with a dermal-based spindle cell neoplasm with infiltrative growth into the subcutaneous tissue, supporting the histopathological diagnosis of dermatofibrosarcoma protuberans.

Macroscopically, the surgical specimen (Figure 5) revealed an en bloc resection of the skin and soft tissue with an irregular external surface and nodular areas corresponding to the lesion. On sectioning, a solid, well-defined tumor with a pale, homogeneous cut surface was identified, infiltrating the subcutaneous adipose tissue without the involvement of the underlying skeletal muscle.

The final diagnosis was dermatofibrosarcoma protuberans, histological grade 1 according to the French Federation of Cancer Centers Sarcoma Group (FNCLCC) grading system. The tumor measured 6 × 4.5 × 3.5 cm. Surgical margins were free of tumor involvement, with the closest lateral margin measuring approximately 0.5 cm. The deep margin, corresponding to skeletal muscle, was also free of neoplasia at 0.5 cm.

The postoperative course was uneventful, with the satisfactory viability of both the muscle and the cutaneous island. Flap viability was assessed clinically using the cutaneous island as a monitoring parameter, evaluating characteristics such as color, capillary refill, and overall tissue perfusion, with no signs of vascular compromise observed. No complications such as infection, dehiscence, or partial flap necrosis were observed. The patient has been followed for four years, with no evidence of local recurrence or distant metastasis to date. Given the achievement of negative surgical margins and the absence of high-risk features, no adjuvant therapy was indicated, and the patient was managed with surgical treatment alone.

## Discussion

DFSP is a low-grade cutaneous sarcoma characterized by a paradoxical biological behavior: while it exhibits minimal metastatic potential, it demonstrates a marked propensity for local infiltration and recurrence. This pattern has been attributed to its tentacle-like subclinical extensions, which may extend several centimeters beyond clinically apparent margins, as described by Allen et al. [11]. This infiltrative growth pattern explains both the high rates of initial misdiagnosis and the risk of recurrence following seemingly adequate resections [11,12].

In this context, the initial misdiagnosis as a keloid in our case aligns with previous reports by Acosta and Vélez, who emphasize that DFSP frequently mimics benign fibroproliferative lesions, including keloids and dermatofibromas [13]. Similarly, Zhou et al. explicitly highlight that DFSP is often clinically confused with hypertrophic scars or benign masses due to its indolent presentation and nonspecific early features [12]. This diagnostic ambiguity contributes to delayed definitive treatment and increases the likelihood of incomplete initial excision.

From a therapeutic standpoint, there is broad consensus that complete surgical excision with histologically negative margins remains the cornerstone of treatment. Both Allen et al. [11] and Acosta and Vélez [13] underscore that the infiltrative nature of DFSP necessitates meticulous margin assessment. However, the optimal surgical approach and margin width remain subjects of ongoing debate.

Classical recommendations for wide local excision (WLE) advocate margins of 2-3 cm, supported by evidence demonstrating improved local control with wider resections. Zhou et al. reinforce this approach, reporting that WLE with 2-3 cm margins, combined with the intraoperative pathological confirmation of negative margins, achieves satisfactory oncologic outcomes in most cases [12]. Furthermore, international guidelines cited by Zhou et al. (including National Comprehensive Cancer Network {NCCN} and European consensus recommendations) reveal variability in margin strategies, ranging from 1-1.3 cm (when complete margin control is feasible) to 2-4 cm in standard practice, reflecting the lack of uniform consensus [12].

Although Mohs micrographic surgery (MMS) offers theoretical advantages through complete margin assessment, its superiority over WLE remains unproven. Zhou et al. emphasize that most comparative data are derived from retrospective series, with no randomized controlled trials establishing the definitive superiority of MMS over WLE [12]. Additionally, MMS is limited by its technical complexity, cost, and restricted availability. Consequently, WLE remains the most widely implemented approach globally, particularly in resource-constrained settings.

Our case reflects this real-world scenario. Despite a closest histological margin of 0.5 cm, which is below commonly recommended thresholds, the patient remains disease-free at four years. This finding may be interpreted considering Allen et al., who suggest that narrower margins may be sufficient when complete tumor excision is achieved and verified histologically [11]. Similarly, Acosta and Vélez emphasize that the key determinant of oncologic control is not solely the margin width but the adequacy of histopathological margin evaluation [13].

Nevertheless, this observation must be interpreted cautiously. Zhou et al. report a recurrence rate of 11.8% in their series using 2-3 cm margins, with recurrence predominantly occurring in recurrent tumors and fibrosarcomatous variants [12]. This is consistent with findings from Marcoval et al. [14] and Saifuddin et al. [15], who report recurrence rates around 11%, reinforcing that tumor biology (particularly fibrosarcomatous transformation and tumor size) plays a critical role in prognosis. Therefore, the favorable outcome in our case likely reflects low-risk tumor biology rather than the validation of reduced surgical margins.

The most significant contribution of this case lies in the reconstructive strategy. DFSP resection frequently results in large soft tissue defects that cannot be closed primarily. Zhou et al. report defects up to 25 × 30 cm, requiring reconstruction in all cases, with 52.9% managed using local flaps and 47.1% with split-thickness skin grafts [12]. Importantly, their results demonstrate that flap-based reconstruction provides superior aesthetic and functional outcomes compared to grafting, with better tissue integration and contour restoration.

This aligns with the principles described by Acosta and Vélez, who advocate for a multidisciplinary approach integrating reconstructive planning into the initial oncologic strategy [13]. Zhou et al. further emphasize that reconstruction should be guided by defect size, depth, vascular supply, and anatomical location, with pedicled axial flaps offering reliable vascularization and adaptability [12]. More recently, reconstructive decision-making has evolved beyond the traditional hierarchical approach, incorporating a more individualized “reconstructive toolbox” concept, in which the optimal technique is selected based on patient-specific and defect-specific factors rather than a fixed algorithm [16].

In this context, the selection of the reconstructive technique must be tailored to balance oncologic safety with functional and aesthetic outcomes. In our case, the use of a pedicled pectoralis major myocutaneous flap exemplifies this approach. Reconstruction was not merely a secondary step but a critical component that enabled adequate oncologic resection while preserving structural integrity and function. Although alternative options such as primary closure, skin grafting, or other local and regional flaps were considered, a myocutaneous flap was selected due to its high reliability, robust vascular supply, and its ability to provide well-vascularized tissue for tension-free coverage [16]. Furthermore, this approach allows improved contour restoration and avoids the contour depression and stigmatizing appearance frequently associated with skin grafts in the anterior chest wall. However, the pectoralis major myocutaneous flap may present certain limitations, including increased bulk and potential donor-site morbidity, which should be considered when selecting the most appropriate reconstructive approach. This concept is reinforced by Zhou et al., who highlight that preoperative imaging (MRI, CT angiography, and ultrasound) is essential for defining tumor extent and guiding both resection and flap design to optimize outcomes [12].

Thus, reconstruction should be conceptualized not as a consequence of tumor excision but as an enabling factor that allows for oncologically sound surgery. The availability of well-vascularized tissue facilitates wider resections when necessary and minimizes postoperative complications. In our case, the absence of complications and durable oncologic control further supports the role of pedicled flaps as a strategic tool in chest wall DFSP.

Finally, certain limitations should be acknowledged. Immunohistochemical analysis was not performed, which precludes additional molecular characterization, such as CD34 expression or *COL1A1*-*PDGFB* fusion; however, the diagnosis was established based on consistent histopathological findings. In addition, histopathological microphotographs were not available due to the retrospective nature of the case, as no images were obtained or archived at the time of the original evaluation. Furthermore, the absence of a standardized long-term aesthetic assessment limits the objective evaluation of reconstructive outcomes. As noted by Zhou et al., small sample sizes and limited follow-up are inherent to most DFSP reports, highlighting the need for larger prospective studies [12].

This case does not challenge current recommendations regarding surgical margins in DFSP but provides clinically relevant evidence supporting the feasibility of achieving durable oncologic control through conventional wide excision combined with immediate reconstruction. More importantly, it highlights that reconstructive surgery is not an ancillary component but a central element in the comprehensive oncologic management of DFSP, particularly in anatomically complex regions and resource-limited settings.

## Conclusions

Dermatofibrosarcoma protuberans may mimic benign lesions, contributing to delayed diagnosis and inadequate initial management; therefore, early histopathological evaluation is essential. Complete excision with negative margins remains the cornerstone of treatment, and reconstructive planning should be considered an integral component of the surgical strategy rather than a secondary step. In this case, a pedicled pectoralis major myocutaneous flap provided reliable, well-vascularized coverage that facilitated adequate oncologic resection, with no evidence of recurrence after four years of follow-up. Despite its inherent limitations, including potential bulk and donor-site morbidity, this case supports an integrated oncologic and reconstructive approach in the management of DFSP.
